# Mask exposure during COVID-19 changes emotional face processing

**DOI:** 10.1371/journal.pone.0258470

**Published:** 2021-10-12

**Authors:** Elyssa M. Barrick, Mark A. Thornton, Diana I. Tamir

**Affiliations:** 1 Department of Psychology, Princeton University, Princeton, New Jersey, United States of America; 2 Department of Psychological and Brain Sciences, Dartmouth College, Hanover, New Hampshire, United States of America; Universita degli Studi di Udine, ITALY

## Abstract

Faces are one of the key ways that we obtain social information about others. They allow people to identify individuals, understand conversational cues, and make judgements about others’ mental states. When the COVID-19 pandemic hit the United States, widespread mask-wearing practices were implemented, causing a shift in the way Americans typically interact. This introduction of masks into social exchanges posed a potential challenge—how would people make these important inferences about others when a large source of information was no longer available? We conducted two studies that investigated the impact of mask exposure on emotion perception. In particular, we measured how participants used facial landmarks (visual cues) and the expressed valence and arousal (affective cues), to make similarity judgements about pairs of emotion faces. Study 1 found that in August 2020, participants with higher levels of mask exposure used cues from the eyes to a greater extent when judging emotion similarity than participants with less mask exposure. Study 2 measured participants’ emotion perception in both April and September 2020 –before and after widespread mask adoption—in the same group of participants to examine changes in the use of facial cues over time. Results revealed an overall increase in the use of visual cues from April to September. Further, as mask exposure increased, people with the most social interaction showed the largest increase in the use of visual facial cues. These results provide evidence that a shift has occurred in how people process faces such that the more people are interacting with others that are wearing masks, the more they have learned to focus on visual cues from the eye area of the face.

## Introduction

In January 2020, reports of a severe novel respiratory illness in the Western United States began appearing in the news. By mid-March the number of COVID-19 cases exceeded 40,000, and local and state governments responded by implementing strict limitations on social gatherings and stay-at-home orders [1]. As evidence emerged in early April that COVID-19 could be transmitted asymptomatically through airborne particles, the Center for Disease Control (CDC) began recommending social distancing practices, where people should keep at least 6-feet apart, and wearing a face covering over the nose and mouth when outside the home [2]. Many Americans were slow to adopt the practice, with only 36% wearing masks in the weeks following the CDC’s announcement [3]. However, as evidence of the effectiveness of face coverings began to mount, mask-wearing practices began to increase. By July, 65% of Americans reported regularly wearing masks outside the home; by August 2020 85% of Americans were wearing masks [4].

This introduction of masks brought with it an enormous shift in the visual social landscape. People could no longer see one of the central percepts in the social realm: others’ faces. This introduction of masks into social exchanges posed a potential challenge—how would people read others’ emotions when a key source of information was no longer available? Here we investigate how this shift in visual input has impacted people’s ability to process emotion in others’ faces.

### Faces as a source of social information

Humans are a social species, and their ability to successfully engage in cooperative interactions is integral to their survival. In order to navigate these interactions, people must utilize information from their environments. One key source of social information is the face. Within the first few minutes of life humans preferentially look at faces, and increasingly seek out and attend to faces in their environment throughout development [5, 6]. As adults, people are more likely to attend to faces than other parts of the body, and spend between 45–60% of the time looking at each other when communicating—and for good reason [7–9].

Faces are a gold mine of social information, and the ability to accurately attend to and perceive them is crucial to social success [10–12]. They allow people to identify familiar others from strangers and provide nonverbal cues during social interactions. For example, the gaze of a social partner signals their attention and engagement in the conversation, while the gaze direction of each party signals whose turn it is to speak [13, 14]. In addition to providing conversational cues, facial expressions allow us to infer the emotions of those around us.

At around 6-months of age infants begin using facial features to discriminate between emotions. By six years old, children attend to discrete facial features when inferring emotions from images [15, 16]. Adult humans are so adept at emotion perception, it only takes 39 ms of exposure to an emotional face for people to perceive and accurately categorize the emotional state [17]. Faces provide so much more than just access to others’ momentary internal states. People also extract a wealth of social information from emotional expressions. For example, people see trait information in others’ emotional expressions. People even process neutral faces as emotional and, in turn, make judgements about dominance, affiliation, and even trustworthiness [18–20]. These judgements have important social implications, as negative traits pulled from emotional expressions can influence people’s willingness to affiliate or cooperate with others.

Together, these studies all highlight the importance of facial cues during social interactions: faces allow us to perceive emotions and make social inferences based on the information we extract. How is our ability to use this information affected if we no longer have access to many of the cues, such as when the people around us are wearing masks?

### The malleability of face perception

The literature offers two possibilities for how masks might impact emotion perception. The first possibility is that face perception will remain intact, and unperturbed. This possibility derives from literature suggesting that face processing is a special, deeply rooted human perceptual ability, and thus unlikely to be impacted by local changes in our sensory environment. Indeed, humans show an innate preference for faces, attending to them with increased specificity within the first few days of life, and even in utero [21, 22]. Over the first months of development, humans rapidly acquire the ability to recognize faces from other objects in dynamic scenes [23]. During this time, infants begin focusing their attention on discrete facial features, such as the eyes and mouth, and use these features to distinguish between mental states [15]. Starting in adolescence, humans move from considering facial features separately and begin considering the face holistically when making judgments about the emotional states of others, using dynamic patterns of eye movements to integrate a combination of facial cues [24, 25]. This ability to recognize emotions and infer emotions from faces improves rapidly throughout childhood, and though the field disagrees on when exactly this ability fully develops, it is generally agreed upon that humans reach expert-level emotion discrimination by late adolescence [26–28]. The findings from this literature suggest that, by adulthood, face perception might have become such an automatic process that the introduction of masks into our environment for a mere five months should not have a significant impact on the strategies we use to derive cues from them.

Alternatively, the literature on learning and plasticity suggests that our perceptual processes should be impacted by shifts in the perceptual data provided by our local environment. The brain actively makes predictions about how things will occur in our environment as we go about our daily lives [29, 30]. When we encounter information that is contrary to our predictions, our brains signal this “prediction error”, and sensory information is passed along so that our brains can update the framework with this new information. The result of this process is knowledge that is not static, but rather continuously updating each day throughout our lives. On a neural level, this updating is reflected in both functional and structural changes in the brain. Several studies using diffusion weighted imaging and diffusion tensor imaging to investigate neural updating have found evidence of learning-induced structural changes in the hippocampus in both rodents and humans [31–34]. Moreover, these changes occur rapidly with short amounts of training, and can be seen in as little as two hours [35, 36].

Research on the neural regions involved in face processing shows evidence of changes across development and context. In adults, face processing is accomplished by two systems: a core system and an extended system [37, 38]. Within the core system, the occipital face area (OFA) first processes and differentiates facial components such as the eyes and mouth [39]. From here, this information is transmitted to the fusiform face area (FFA), which is crucial for face identification, and the posterior superior temporal sulcus, which tracks dynamic facial features and movements that are important for social cue identification and emotion recognition [40–46]. Neuroimaging studies show that the FFA and ventral temporal cortex continue to develop well into adulthood with increasing specificity [47–49]. Infants have similar spatial organization of the temporal and parietal cortices as adults, but still respond differently to stimuli than adults, suggesting that these regions continue to be refined over time [50]. The extended system works with the core system to extract higher level social information [51–54]. Environmental changes, such as a persistent increase in stress, have been shown to cause synaptic plasticity within this extended face processing system [55, 56].

Behavioral research in this domain provides further evidence for the malleable nature of face-processing. A recent large-scale study examined age-related changes in face processing and found evidence that this skill continues to develop much later than previously thought [57]. In three online experiments, participants 10–70 years old completed a series of face recognition tasks where they learned a set of novel faces and later identified them from a set of stimuli. Results revealed that the ability to learn and recognize new faces improves into the 30s, rather than peaking in adolescence. Together this literature suggests that our brains and behavior adapt as our environment changes. Thus, in an environment with pervasive mask-wearing, people may indeed rapidly shift in how they process faces.

In response to this significant environmental change, recent research has begun to examine the impact of masks on face perception. Studies specifically examining how masks impact our face processing have found evidence that face masks lead to a decrease in face recognition accuracy and impair face matching for both familiar and unfamiliar faces. For example, a recent study looking at discrete emotion identification in children found that accuracy was lower for faces that were wearing masks [58]. Further findings suggest that masks may also lead to less holistic processing of faces [59, 60]. These studies provide compelling evidence that the introduction of masks into our social environment may be impacting our face processing abilities and strategies.

Our current work offers two advantages to these earlier findings on changes in face processing. First, the most recent studies have focused on processing neutral faces. However, many of the interactions people have day to day require the identification of others’ emotions. Here we focus specifically on the processing of emotional faces. Second, here we evaluate the long-term effects of masks on face processing. Earlier studies examined how people processed faces that were *currently* wearing masks. We examine how people process faces *without* masks after months of mask exposure. If people adapt to masks by changing how they approach facial emotion perception, this offers deep evidence for the pervasive impact of masks on our social cognition.

### Current studies

In two studies, participants completed an emotion perception task where they had to assess the emotional similarity between a pair of unmasked faces. We measured how participants used two types of information to make their responses: First, participants could use purely visual cues, referring to the placement and shape of facial landmarks, such as eyebrows, nose, and mouth. For example, participants who used this type of cue might rate faces as similar if both pairs of eyes were opened wide, eyebrows were raised, and mouths were downturned. Second, participants could use affective cues, referring to the valence and arousal expressed by the faces. Participants who relied on this type of cue would rate faces as similar if they were both expressing negative emotions, such as disgust and anger, or dissimilar if they were expressing emotions of differing intensities, such as sleepy and surprised.

People use both affective and perceptual cues when identifying facial emotion expressions [61, 62]. Perceptual cues—the configural and morphological features of a face—may aid early emotion identification, while affective cues—the valence or arousal expressed by a face—may play a more significant role in emotion recognition [see [63] for a comprehensive review]. Our current design allows us to evaluate both local and holistic face processing by comparing whether participants use facial components (visual cues) versus more conceptual cues from the face as a whole (affective cues) when making emotion judgements.

In Study 1 (August 2020), participants also answered a series of questions about the use of masks in their community and their recent social activities, allowing us to test how individual differences in mask exposure impacted the facial information they used. In Study 2, participants completed the emotion perception task at two time points: prior to widespread mask adoption, in April 2020, and then again five months later after widespread mask adoption, in September. Participants in Study 2 also answered questions about community mask use and their recent social activities at the second time point (September 2020), allowing us to test how the accumulation of mask exposure impacted the facial information they used.

## Study 1

### Methods

#### Participants

Participants (*N* = 200; 86 Female, 20–70 years old, M_age_ = 39) were recruited through Amazon’s Mechanical Turk (mTurk) online platform to complete a 20-minute study for $1.60 at the end of August 2021, approximately 4 months after the CDC recommended mask-wearing practices. A target sample size of 191 was determined based on a pilot study (see S3 Appendix for pilot study details), using a power analysis targeting an effect size of *f* ^2^ = .09 for the effect of mask exposure on cue use, significance level of 0.05, and power of 0.95. Participation was restricted to mTurk workers in the United States with > 95% approval ratings. Participants were excluded a priori if they reported that an English comprehension less than “Good” (N = 0). The Institutional Review Board at Princeton University approved this study and all following studies. All participants provided informed consent through online consent form.

#### Emotion similarity task

**Fig 1:**
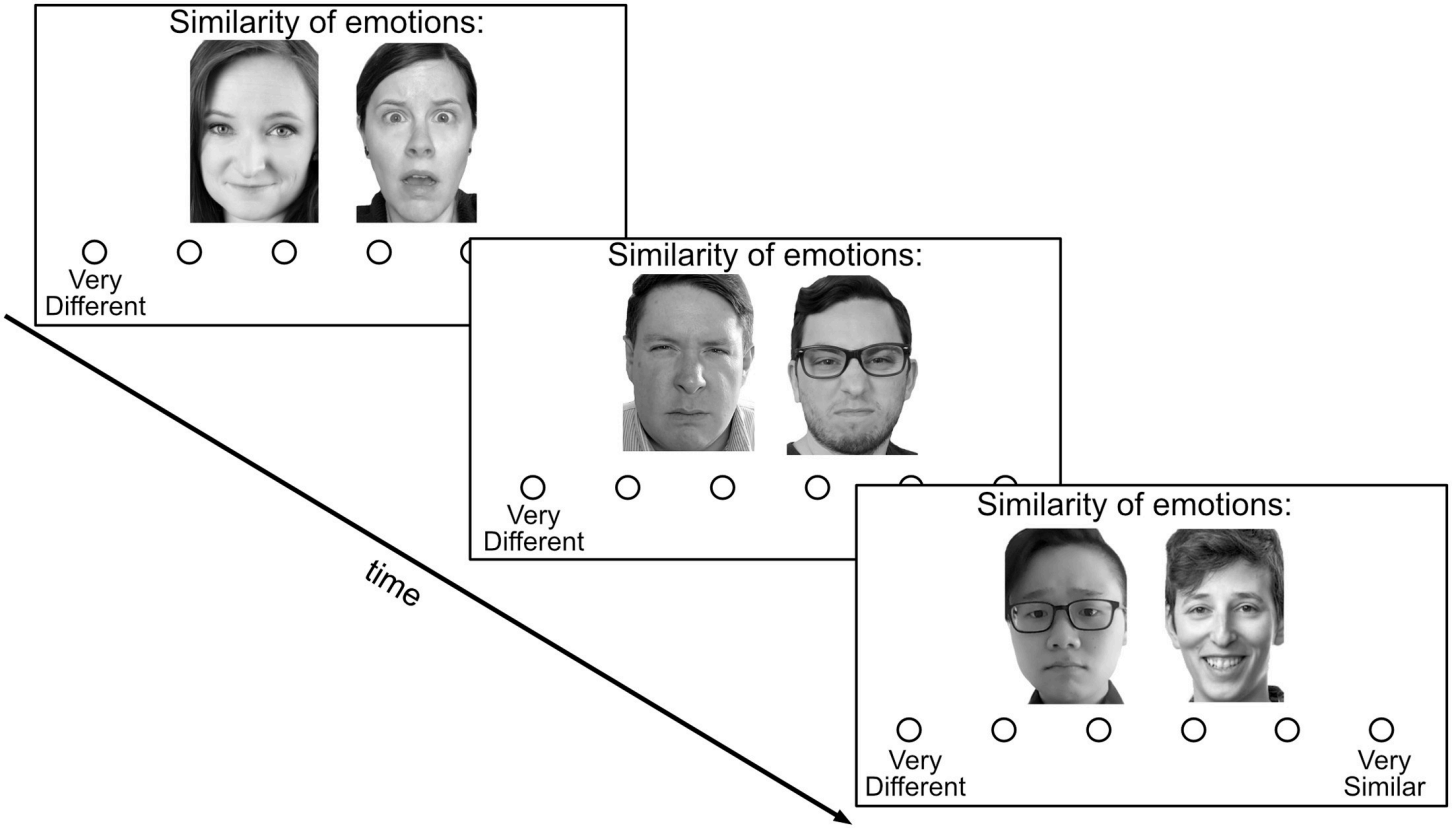
Example trials for face similarity task. Each trial presents a pair of emotional faces. Participants rate the similarity of the expressed emotions. The images pictured here are similar, but not identical, to the original images used in the study, and are for illustrative purposes only.

Our primary goal in this study was to assess individual differences in emotion perception from faces, after varying levels of mask exposure. To measure emotion perception, participants completed a task online in Qualtrics Survey Software [64], in which they rated the similarity of emotions expressed on a pair of faces. On each trial of this task, participants were presented with two faces, side by side. Participants were instructed “For each pair of images, you will rate the similarity of the two emotions. You will make your rating on a scale from ‘very different’ to ‘very similar’”. Ratings were made on 6-point scale (Fig 1). Participants had an unlimited amount of time to respond to each pair. This task allowed us to examine participants’ perception of emotions without conceptual influence, as participants were not required to use language, or engage in discrete emotion categorization in order to rate the similarity of the faces. Participants rated a total of 45 pairs of faces, each expressing one of 7 emotional states (anger, contempt, disgust, fear, happy, sad, surprise). Pairs of faces were presented in randomized order.

#### Task stimuli

**Fig 2:**
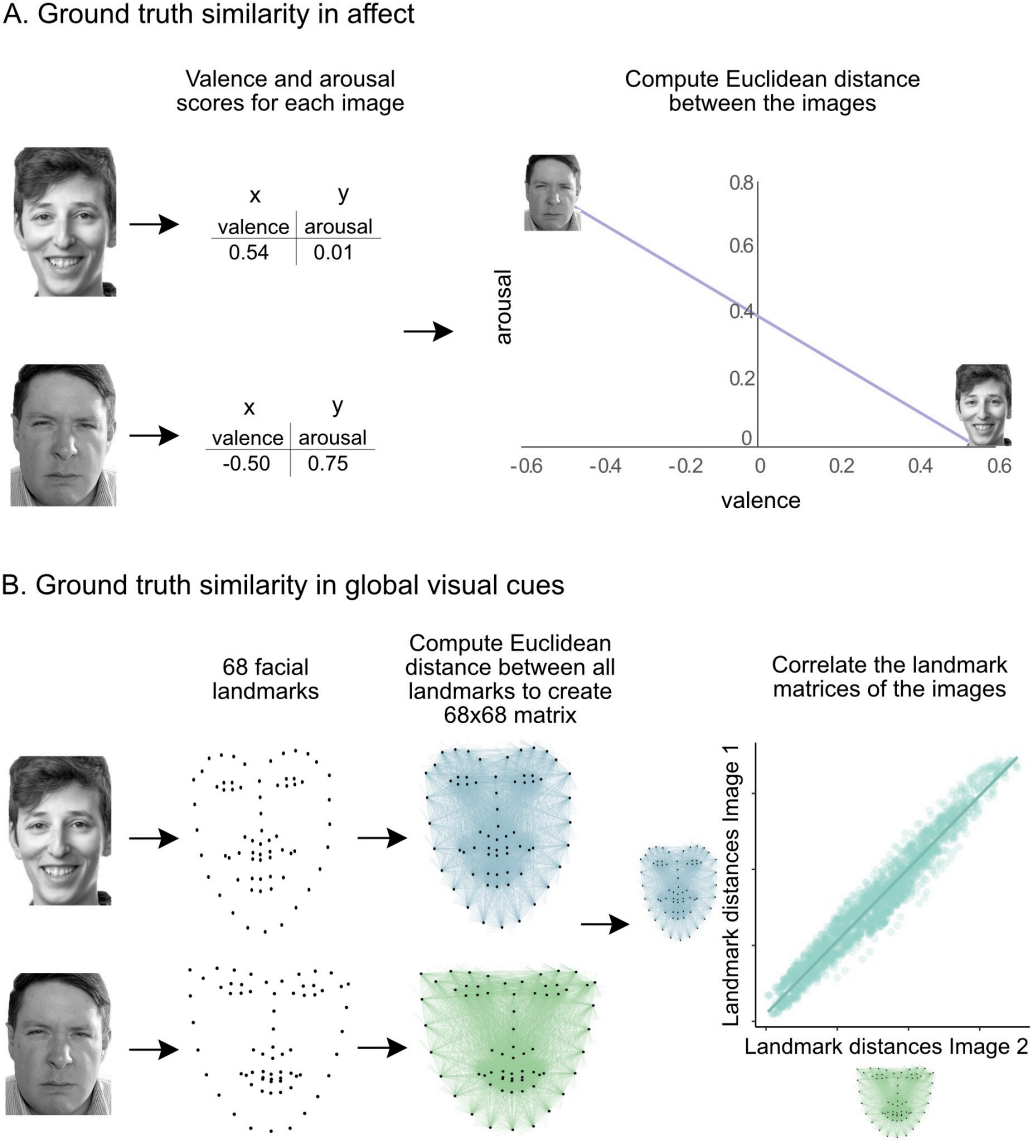
Ground truth calculations. A) The ground truth affective similarity is based on valence and arousal ratings. These ratings served as x-y coordinates for each face. The affective similarity score was calculated as the Euclidean distance between these values. B) The ground truth visual similarity is based on the Euclidean distances between all 68 facial landmarks. These landmarks were obtained from automated software (ResNext) for each face. Distances between each landmark were represented in a 68x68 matrix for each image. The global visual similarity score was calculated as the correlation between these two vectorized matrices.

**Fig 3:**
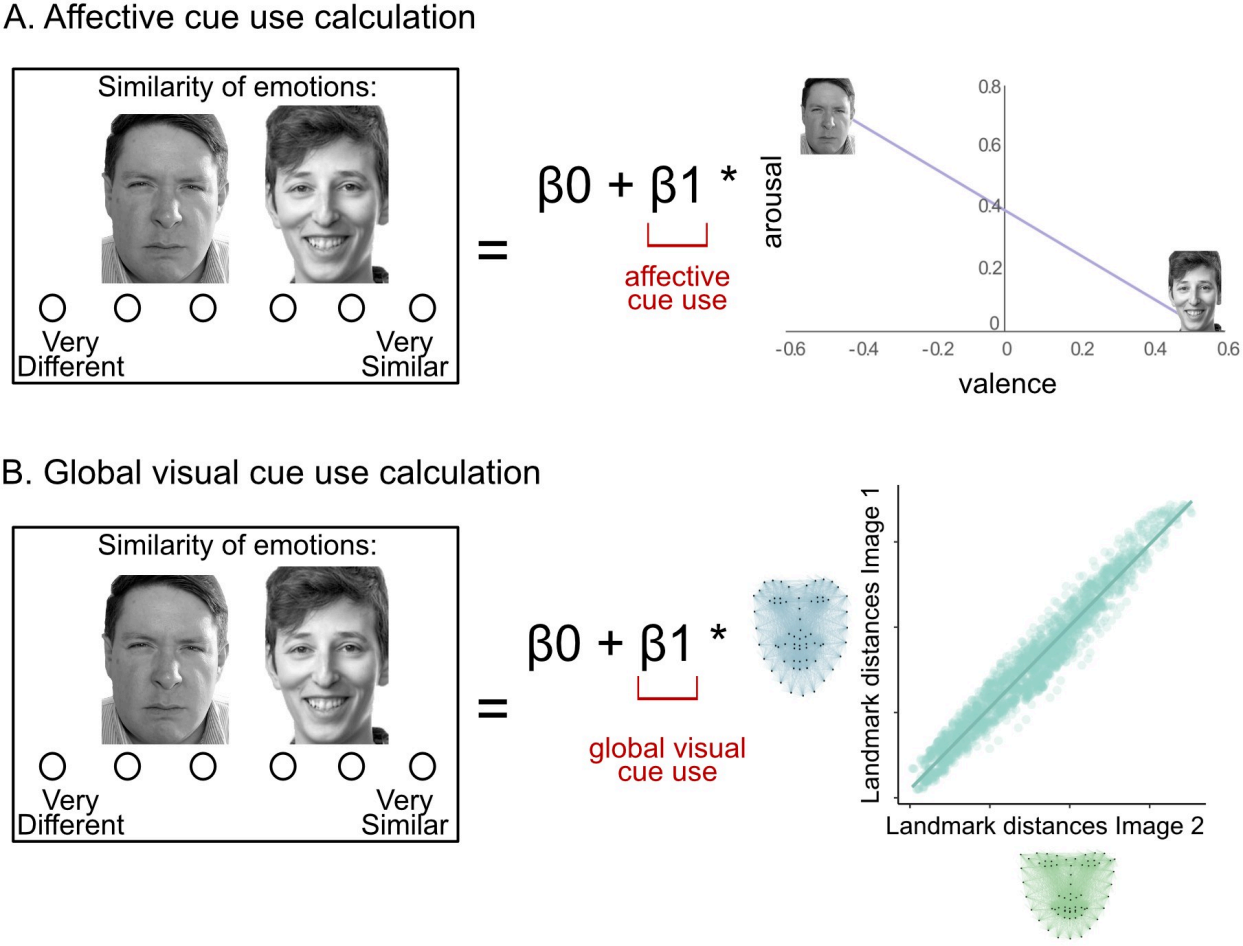
Cue use calculations. The participants’ similarity ratings were predicted by A) the affective similarity score, to calculate the affective cue use score, and B) the visual similarity score to calculate the global visual cue use score.

The individuals pictured in Figs 1–3 have provided written informed consent to publish their images alongside the manuscript. The images pictured in the figures are similar, but not identical, to the original images used in the study, and are for illustrative purposes only. Faces used as task stimuli were obtained from AffectNet, a database of emotional facial images collected from the internet [65]. Images were manually selected from 18,974 files to 1) remove famous individuals, 2) remove poor quality images, 3) pare down to images where the subject was facing the camera and the full face was visible, 4) represent a broad range of races, genders, and ages, for a final sample of 98 images, 14 from each of 7 discrete emotion categories. These images were then converted to grey scale with similar luminance and contrast. Next, we used the visual and affective similarity values in a greedy search algorithm to 1) generate pairings with a uniform distribution of both visual and affective similarities using a Kolmogorov–Smirnov test, and 2) minimize the visual and affective correlations across pairs, resulting in 49 pairs of faces. Data from a pilot of these 49 pairs was then used to calculate task reliability. We calculated the contribution of each image pair to reliability by recalculating split-half reliability 49 times using a leave-one-out procedure. This resulted in a final set of 45 pairs of images with an internal consistency estimate of 0.75, based on an odd-even split-half reliability correlation. This final sample contained 13 anger, 12 contempt, 14 disgust, 14 fear, 12 happy, 13 sad, 12 surprised faces. Pairings included faces expressing both the same, as well as different discrete, emotions. Pairs were uploaded to Qualtrics at an overall size of 612x275 pixels.

In addition to providing images, the AffectNet Database provided information about both the affect expressed in each face, as well as the visual landmarks present in each face. We used this information in order to calculate the affective and visual similarity of each pair of faces, respectively (Fig 2).

First, expert annotators provided ratings of the valence and arousal of each face stimulus. Twelve annotators were trained to rate visual examples on the continuous dimensions of valence and arousal, defined based on the circumplex model. Valence was defined as a dimension with positivity at one pole, and negativity on the other; arousal was defined as a dimension with exciting/agitating at one pole and calm/soothing at the other. Annotators provided a single valence and arousal rating for each face; these values were averaged across all annotators. For each face pair, we could calculate the extent to which they were objectively similar in terms of these affective cues (Fig 2A). We calculated the ground truths for affective similarity using these valence and arousal ratings. Each face had a single value for valence and arousal. We calculated the *valence cues* as the absolute difference between valence ratings for each face, and *arousal cues* as the absolute difference between arousal ratings for each face. We calculated *affective cues* as the Euclidean distance between the valence and arousal ratings for pairs of faces by using the valence as the x-coordinate, and arousal as the y-coordinate.

Second, we extracted purely visual information for each stimulus by using the x-y coordinates of 68 facial landmarks for each face. These landmarks were automatically annotated by the AffectNet research group using ResNext Neural Network [66]. For each face pair, we could calculate the extent to which they were objectively similar in terms of these visual cues (Fig 2B). We calculated the ground truths for visual similarity between pairs of images by comparing the locations of the 68 facial landmarks. Within each face, we first computed the Euclidean distances between each pair of landmark points using the coordinates provided from AffectNet. This resulted in a 68x68 matrix with all of the relative distances between all landmarks for each face. We correlated those distances across different faces to estimate their visual similarity. We calculated three visual cue metrics: (i) *global visual cues*, using all 68 facial landmarks, (ii) *eye cues*, using all 26 landmarks above the nose, and (iii) *mouth cues*, using all 42 landmarks below the eyes.

These values served as the ground truth similarity for each of six types of cues: global visual, eye, mouth, affective, valence, and arousal. We could then compare these scores to participant ratings to determine how much they used each type of information in their similarity judgements. To calculate a use score for each type of cue (global visual, eye, mouth, affective, valence, arousal) for each participant, we regressed their standardized similarity rating, across all 45 ratings, onto the ground truth score for each cue type (Fig 3). Previous studies have utilized similar statistical procedures by regressing the results of individual differences representational similarity analyses onto facial expression data [67].

#### Mask and social interaction

To examine the effect of mask exposure on emotion perception, participants answered five questions, with seven specific sub-questions about mask use in their household and community (e.g., “What percentage of time do you personally wear a mask while engaging in activities outside of the home?”; “Report the percentage of people wearing masks while you were Interacting with others face-to-face over the *last 24-hours*”; see S2 Appendix for complete list of questions). The questions were z-scored and combined into a composite measure of mask exposure (α = 0.71). Cronbach’s α was computed using the *alpha()* function of the psych (v1.9.12.31) package. We conducted separate simple linear regressions to predict each cue (global visual, eye, mouth, affective, valence, arousal) as a function of mask exposure using the *lm()* function of the stats (v4.0.2) package. Standardized betas were extracted from the models using the *lm*.*beta()* function of the lm.beta (v1.5.1) package. This and all analyses were conducted using RStudio (v1.3.959) [68].

Though we hypothesize that mask exposure may have an impact on how people are using facial cues, we recognize that individual differences in social activity engagement may impact the effect of mask exposure on participants, even if their community generally wears masks. That is, more social interaction should increase mask exposure, thus magnifying the effects of mask exposure, while less social interaction, even if most of the community are wearing masks, should minimize the impact of mask wearing on perception. To examine the mediating effect of social interaction on cue use, participants answered 8 questions about the number of hours spent in social interaction and the number of people engaged with over the past 24-hours and past month (e.g., “Around other people in the same physical space, but not interacting with them”; Interacting with others face-to-face”). The responses were heavily skewed, and therefore log-transformed. They were then z-scored and combined into a single composite measure of social interaction (α = 0.82).

Additionally, for exploratory purposes, participants answered four questions, with six specific sub-questions about the number of hours spent, and the number of people engaged with in indirect social activities over the past 24-hours and past month (e.g., “Talking to one or more people on video calls such as Skype or FaceTime”; “Watching TV shows or movies”). The questions were log-transformed and z-scored, then combined into a composite measure of virtual social interactions (α = 0.80; See S2 Appendix for complete list of questions and S1 Appendix for results from this analysis). In addition to examining the mediating effect of social interaction, we also examined the impact of social interaction alone on cue use (See S1 Appendix—the effects of social interactions—for results from this analysis).

### Results

**Fig 4:**
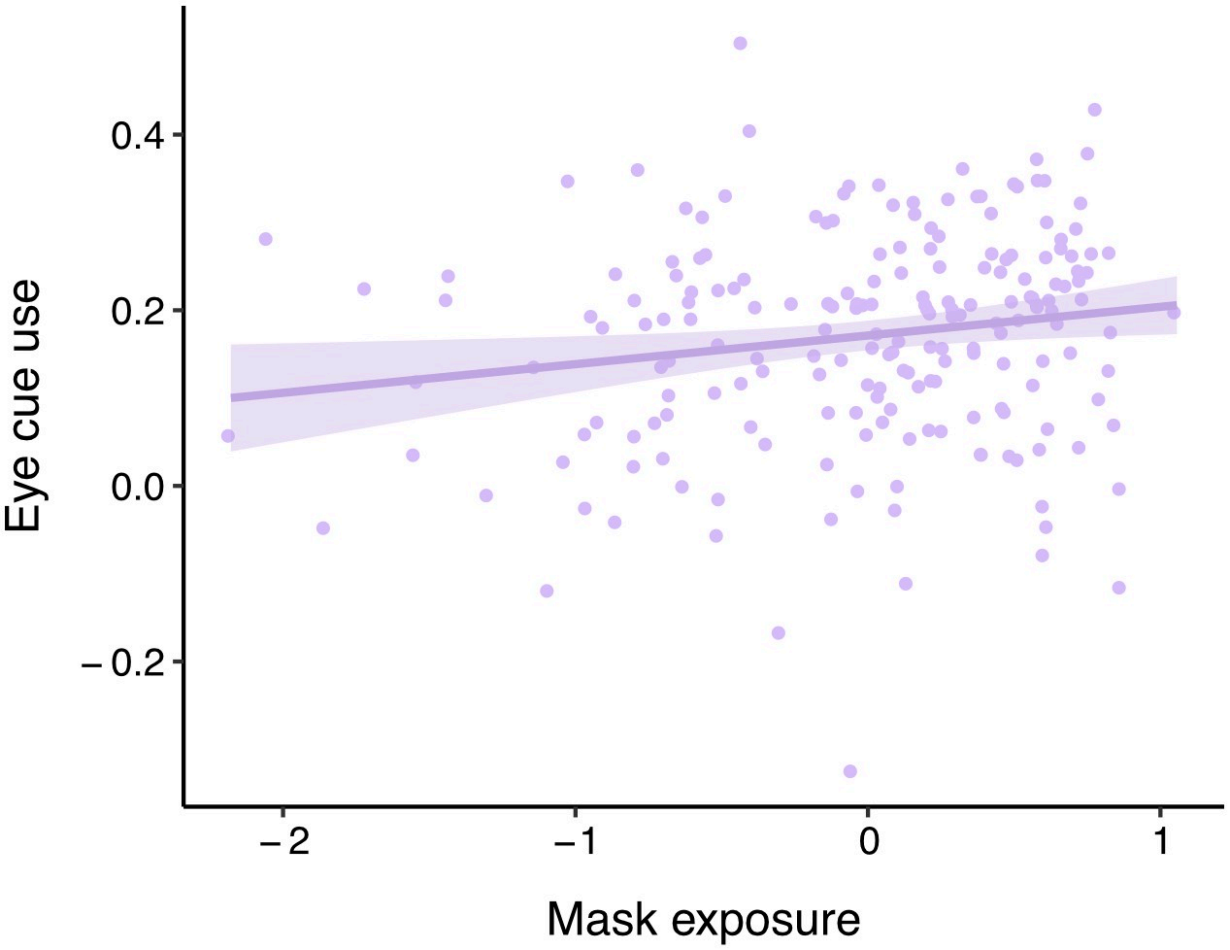
Effect of mask exposure on eye cue use in Study 1. Use of eye cues in emotion judgements increased as exposure to masks increased. Transparent band reflects 95% confidence interval. Created using ggplot2 (v3.3.0) in RStudio.

Our primary analyses tested whether mask exposure predicted participants’ use of different facial cues in their ratings of emotion similarity. Results revealed that mask exposure significantly predicted an increase in global visual cues (*β* = 0.14, *p* = 0.04). This effect was specific to the eye cues (*β* = 0.17, *p* = 0.01; Fig 4), and not mouth cues (*β* = 0.10, *p* = 0.18). Mask exposure did not have a significant impact on use of affective (*β* = 0.18, *p* = 0.27), valence (*β* = 0.07, *p* = 0.34), or arousal cues (*β* = 0.04, *p* = 0.60; see Table A in S1 Appendix for full model statistics). Similar results were obtained when controlling for the age of the participants (See S1 Appendix).

Next, we tested whether exposure to masks affected cue use differently depending on the amount of social interaction people engaged in. We conducted six regressions with each cue (global visual, eye, mouth, affective, valence, arousal) as the DV and the interaction of mask exposure and social interaction as the predictor. Results showed no significant effect of the interaction between mask exposure and social interaction on any of the cue types (eye: *β* = -0.05, *p* = 0.46; mouth: *β* = -0.03, *p* = 0.68; global visual: *β* = -0.06, *p* = 0.40, affective: *β* = -0.01, *p* = 0.89; valence: *β* = -0.01, *p* = 0.89, arousal: *β* = 0.03, *p* = 0.66; see Table C in S1 Appendix for full model statistics).

### Discussion

Study 1 provided evidence that use of global visual cues, and eye cues more specifically, increase as people are exposed more to others wearing masks. This offers preliminary support for the hypothesis that something as fundamental as emotion perception may not be an immutable ability. Instead, the way we process faces may depend on the data that we continually take in about faces, even into adulthood. As data from the lower half of people’s faces decreases due to mask wearing, people seem to focus more on the eye region in order to make judgments about emotions, even when information about the whole face is available.

## Study 2

The results of Study 1 provided initial evidence that mask exposure changes the way people process faces by increasing their use of visual facial cues, particularly in the eye region of the face. In Study 2, we used a longitudinal design that was preregistered after time 1, but prior to time 2 data collection (https://osf.io/7dx3p) to assess changes in cue use, specifically eye cue use, between two time points. We also examined whether mask exposure explained these differences. We expected that eye cue use will have increased from time 1 (April 2020) to time 2 (September 2020). We also continued to examine differences in mouth, global visual, arousal, valence, and affective cues between the two time points, and further explored whether mask exposure and the interaction between mask exposure and social interaction explained any changes in cue use.

### Methods

Participants in Study 2 completed the emotion similarity task at two time points: prior to widespread mask adoption, and after five months of varying levels of mask exposure. We used the emotion similarity task to assess changes in participants’ use of six types of face cues (global visual, eye, mouth, affective, valence, arousal) when making emotion perception judgements across the two timepoints. To test this, we first computed cue use scores at both time 1 and time 2 for each participant. Next, we conducted paired samples t-tests on each cue use score between time 1 and time 2. Effect sizes were computed using the *cohensD()* function of the lsr package (v0.5).

Participants also completed the mask and social interaction surveys from Study 1 at time 2 only. As in Study 1, responses from both measures were heavily skewed and log-transformed prior to being z-transformed and combined into their respective composites. These surveys allowed us to test how interim mask exposure impacted any changes in the information participants used to make emotion perception judgements, and if this effect of mask exposure differed depending on the amount of social interaction people engaged in.

#### Participants

Participants first completed the emotion similarity task in April 2020 (time 1 N = 240) using a Qualtrics online survey. Participants were recruited through Amazon’s Mechanical Turk (mTurk) online platform. Participation was restricted to mTurk workers in the United States with > 95% approval ratings. These same participants were re-contacted in September 2020 through mTurk and informed of their eligibility for a new study. Participants were recruited to complete the 20-minute emotion similarity task again for $4.00 using a Qualtrics online survey. Data collection ceased after a one-week period. All participants who completed the emotion similarity task at time 2 (N = 146; 61 Female, 18–74 years old, M_age_ = 38.33) were included in our analyses. Participants were excluded at time 1 due to poor English comprehension (N = 1) or not completing a full task in the study (N = 0).

### Results

**Fig 5:**
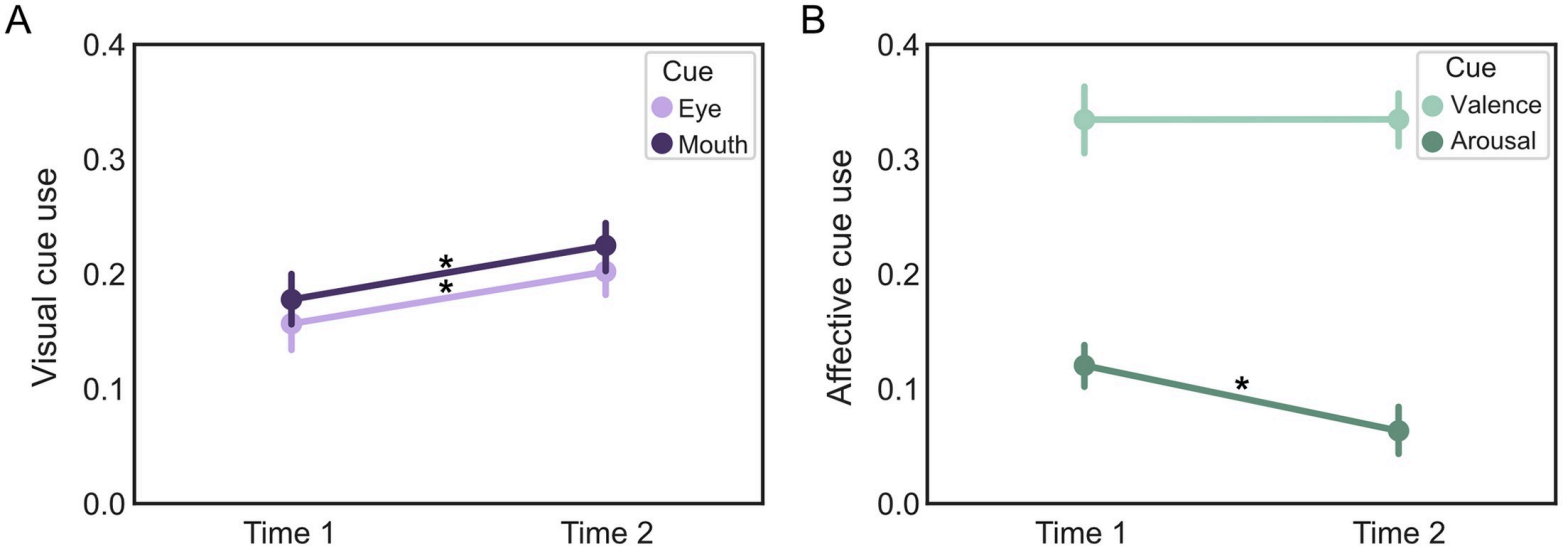
Change in facial cue use between time 1 and time 2 in Study 2. A) Eye and mouth cues use increased from time 1 to time 2. B) Arousal cue use decreased from time 1 to time 2. * *p* < 0.001. Error bars represent 95% confidence intervals. Created using the seaborn package (v0.10.0) for Python (v3.7.6).

In our primary analyses, we examined whether cumulative mask exposure over the course of five months increased the extent to which people rely on visual and affective face cues when making emotion perception judgments. As expected, results showed that people used global visual cues to a greater extent at time 2 compared to time 1 (*t*(145) = 4.49, *p* < 0.001, *d* = 0.37, CI:[0.03, 0.08]; Fig 5). This increase in global visual cues resulted from increase in use of both eye cues (*t*(145) = 3.61, *p* < 0.001, *d* = 0.30, CI: [0.02, 0.07]) and mouth cues (*t*(145) = 4.02, *p* < 0.001, *d* = 0.33, CI: [0.02, 0.07]). In addition, we found evidence that people used arousal cues to a lesser extent at time 2 compared to time 1 (*t*(145) = -4.81, *p* < 0.001, *d* = 0.40. CI: [-0.08, -0.03]). There were no significant differences between timepoints for valence (*t*(145) = 0.01, *p* = 0.99, CI: [-0.03, 0.03]) or affective cues (*t*(145) = -1.13, *p* = 0.26, CI: [-0.04, 0.01]).

Next, we tested whether exposure to masks between time 1 and time 2 could account for the difference in cue use between time 1 and time 2. To test this, we first computed difference scores for each cue type, subtracting time 1 scores from time 2 scores, such that positive scores reflect higher cue use at time 2 and negative scores reflect higher cue use at time 1. We then conducted separate simple linear regressions with each difference in cue use as the DV predicted by mask exposure. Results showed that the difference in cue use between time 1 and time 2 could not be explained by mask exposure alone (eye: *β* = -0.05, *p* = 0.51; mouth: *β* = 0.005, *p* = 0.95; visual *β* = -0.008, *p* = 0.92; arousal: *β* = -0.11, *p* = 0.18; valence: *β* = -0.004, *p* = 0.96; affective: *β* = -0.02, *p* = 0.82; see Table E in S1 Appendix for full model statistics). We also conducted separate simple linear regressions with each difference in cue use as the DV predicted by social interaction alone (See S1 Appendix for results from this analysis).

Finally, we examined whether mask exposure would impact participants differently depending on the amount of social interaction they engaged in. To test this, we regressed the interaction between mask exposure and social interaction onto the difference in cue use, separately for each cue type. The interactions were probed using the *sim_slope()* function of the interactions package (v1.1.3) by testing the conditional effects of mask exposure at 3 levels of social interaction: at the minimum value, the mean, and the maximum value.

**Fig 6:**
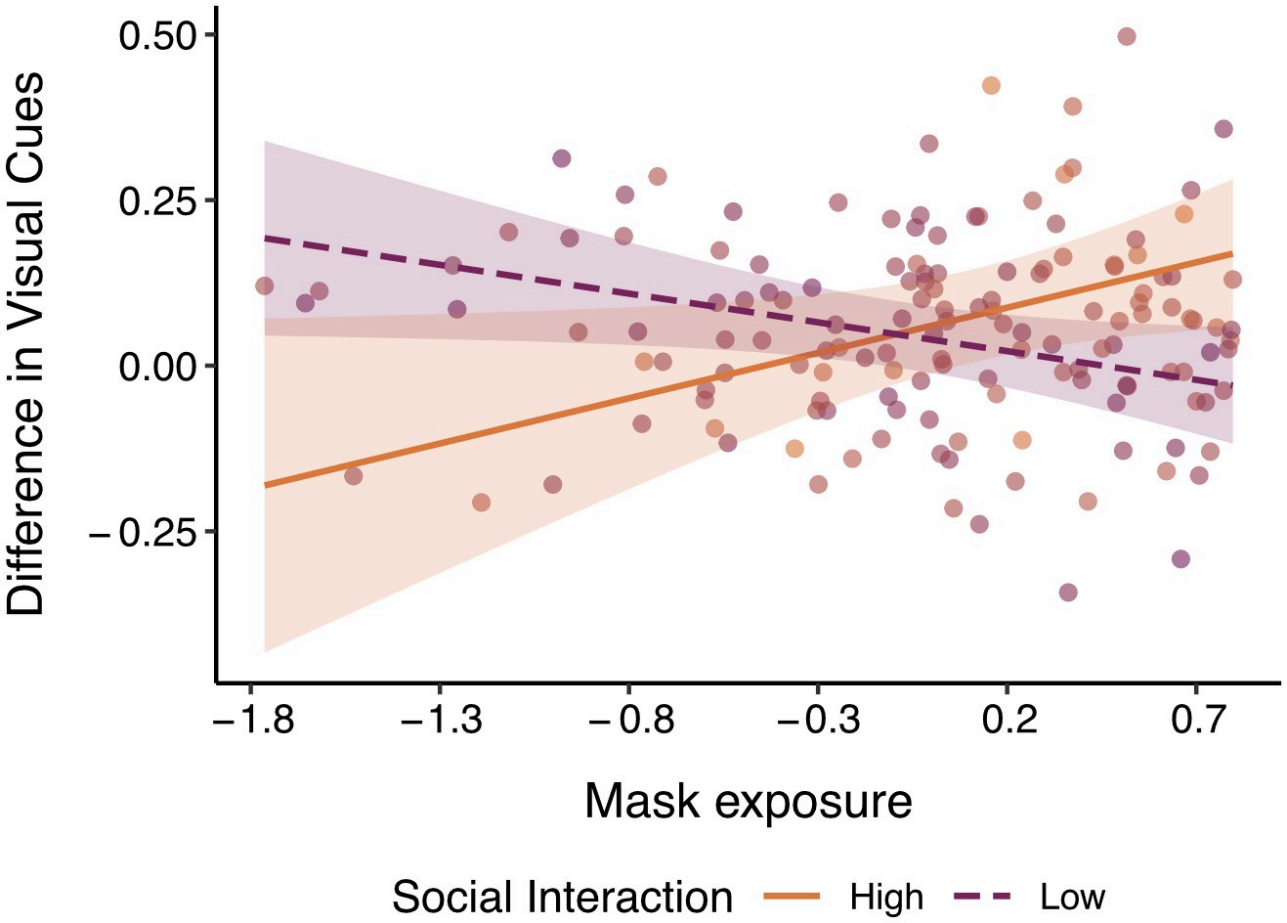
Effect of mask exposure on global visual cue use as a function of social interaction in Study 2. Social interaction mediated the relationship between mask exposure and visual cue use between time 1 and time 2. People with the least amount of social interaction had the greatest decrease in global visual cue use, and people with the most amount of social interaction had the greatest increase in use of global visual cues. Transparent bands reflect 95% confidence intervals.

Results revealed a significant interaction for global visual cues, such that the effect of mask exposure changed at different levels of social interaction (*β* = 0.19, *p* = 0.02; Fig 6.; see Table G in S1 Appendix for model statistics of all interactions). Analysis of the simple effects showed that for people with the highest levels of social interaction, use of global visual cues increased as mask exposure increased (*b* = .14, *p* = 0.04); for people with the least social interaction, use of global visual cues decreased as mask exposure increased (*b* = -.09, *p* = 0.04). There was no effect of mask exposure on people with average social interaction for global visual cues (*b* = .003, *p* = 0.90; see Table H in S1 Appendix for model statistics of all simple effects).

These effects were specific to eye cues, which showed a similar pattern of results to global visual cues in general. There was a significant interaction (*β* = 0.19, *p* = 0.02) such that the effect of mask exposure increased use of eye cues for individuals with the highest level of social interaction (*b* = .13, *p* = 0.06); people with the least social interaction actually showed a decrease in the use of eye cues (*b* = -.10, *p* = 0.02). There was no effect of mask exposure on people with average social interaction (*b* = -0.01, *p* = 0.70). Similar results were obtained when controlling for the age of the participants (see S1 Appendix). There were no interaction effects between mask exposure and social interaction for mouth cues (*β* = 0.13, *p* = 0.11), affective cues (*β* = -0.006, *p* = 0.94), valence cues (*β* = -0.01, *p* = 0.91), nor arousal cues (*β* = -0.002, *p* = 0.98).

Finally, we ran exploratory analyses that deviated from those outlined in the preregistration, to mirror the analyses run in Study 1. We examined the effects of mask exposure on participants’ use of facial cues at time 2. We tested this by regressing mask exposure onto each cue type (eye, mouth, visual, valence, arousal, affective) separately. We found that mask exposure did not significantly predict a change in eye (*β* = 0.07, *p* = 0.43), mouth (*β* = 0.07, *p* = 0.37), global visual (*β* = 0.09, *p* = 0.25), affective (*β* = 0.09, *p* = 0.26), valence (*β* = 0.10, *p* = 0.21), or arousal cues (*β* = -0.05, *p* = 0.52). We also examined whether exposure to masks affected cue use differently depending on the amount of social interaction people engaged in. We tested this by conducting separate regressions predicting each cue use (eye, mouth, visual, valence, arousal, affective) from the interaction between mask exposure and social interaction at time 2. Results revealed a significant interaction for global visual cues, such that the effect of mask exposure changed at different levels of social interaction (*β* = 0.19, *p* = 0.02). Analysis of the simple effects showed that for people with the highest levels of social interaction, use of global visual cues increased as mask exposure increased (*b* = .15, *p* = 0.01). There was no effect of mask exposure on people with average social interaction (*b* = .003, *p* = 0.09), or those with the least amount of social interaction (*b* = -.05, *p* = 0.21). There were no significant interactions for eye (*β* = 0.12, *p* = 0.16), mouth (*β* = 0.15, *p* = 0.08), affective (*β* = 0.06, *p* = 0.50), valence (*β* = 0.04, *p* = 0.65), or arousal cues (*β* = 0.03, *p* = 0.72).

### Discussion

The primary findings from Study 2 extend the results from Study 1, demonstrating that the more people are exposed to others in masks, the more they rely on visual cues when perceiving emotions. We see an increase in this reliance on visual cues when comparing performance from before mask wearing was widespread to performance five months after mask wearing practices have increased. People who are engaging in the most social interaction appear to have the greatest increase in use of visual cues.

In our exploratory analyses, we find that the main effect of mask exposure at time 2 differed in significance from the analogous analysis from Study 1. In Study 1, participants with more mask exposure relied on global visual and eye cues to judge emotions. However, we find parallel results in the interaction effects from time 2, where individuals with more mask exposure and social interaction relied more on global visual cues. This finding is mirrored in our longitudinal Study 2 analyses, where individuals with more mask exposure and social interaction show an increase in visual cue use from time 1 and time 2.

Together, the findings from these studies indicate that people are adapting to the information that is most available in their environment. In the case of a world where social interaction takes place with half of the face obscured by masks, people increasingly attend to the eye region of the face when making emotional judgements.

## General discussion

As more evidence emerges that social distancing and mask wearing are the best protective measures against COVID, people are increasingly exposed to faces that are partially occluded by masks. Interacting with people wearing masks is an enormous shift from our social norm. Are masks impacting our ability to perceive emotion from faces? Across two studies we provide evidence that a significant shift has occurred in how people process emotions from faces: people are focusing on the visual cues to a greater extent, particularly the information around the eyes. Further, people seem to be focusing *less* on the actual affective information depicted in the face, particularly the cues to arousal. This shift occurred despite task instructions to make similarity judgements based on the emotion expressed in the faces, for which attending to the affective cues of the face should have been more relevant than attending to the visual features.

In Study 1, we see a main effect of mask exposure, such that greater exposure to masks means increased attention to the eye area. Study 2 assessed the effects of mask exposure by comparing performance on a face processing task from before and after mask-wearing became the norm in the United States. People in this study showed greater use of both eye and mouth cues and a decrease in arousal cues after five months of mask exposure, compared to before. Both studies consistently show an increase in the extent to which people are relying on eye cues, as well as global visual cues, to make emotion judgements. The two studies differ in the significance of the mouth cues, though the results in Study 1 still indicated a trend in a positive direction (*β* = 0.10). In addition to observing these effects of masks overall, we also observed an interaction between mask exposure and social interaction in Study 2, such that people with more social contact with individuals wearing masks show the largest shift in face processing. This finding did not emerge in Study 1. Any differences in significance across the two studies are likely attributable to differences in design and power: Study 1 examined mask exposure at a single timepoint *across* participants, whereas Study 2 examined changes in mask exposure over time *within* participants. These change scores calculated in Study 2 may have removed unrelated heterogeneity in cross-sectional mask exposure ratings, thus allowing this effect to be detected.

We conducted this study with an initial hypothesis that there would be a shift in how people are processing emotions with the introduction of masks into their environment. However, we remained agnostic about *how* the change would materialize. Why are we observing an increase in visual cues but a decrease in arousal cues? One possibility for the increase in use of eye cues is that people are shifting to use the visual information that is most readily available to them. With masks covering the lower half of the face, people are increasingly relying on the eyes to infer others’ emotions during the majority of their social interactions. This reflects a shift from typical face processing, in which people process both neutral and emotional faces using a dynamic pattern of eye movements to process expressions in a more holistic manner [24, 25, 69] These results are also in line with a recent study examining the impact of masks on face processing, which found that viewing masked faces resulted in a reduction in holistic face processing, and a shift to relying on the available facial features [59].

Indeed, as people shift their focus to the *position* of facial features, they do so at somewhat of a cost to the conceptual information present in the face as a whole. Specifically, we see the consequences of this shift in attention in our affective cue results: people are using arousal cues less. This shift to focusing on the physical locations of features rather than a more holistic view might also explain why we see an increase in mouth cues, despite the mouth largely being covered by masks during social interactions. If people are learning to shift to using the physical placement of facial components rather than more conceptual cues of the face as a whole, they might then have an increased sensitivity to the position of separate facial components and direct their attention to them when they are available. Another possible explanation for the decrease in arousal cues is that there may be a limit to the amount of facial information people process at any given time. If so, then focusing on the eye area of the face may detract from focus on arousal cues. To fully understand how face processing may be changing, future research would benefit from eye-tracking to confirm where people are attending as they observe emotional faces.

These findings support the hypothesis that humans are highly adaptive to the statistics of their environment. So much so that even skills thought to be fully developed early in life can still adjust in new environments, well into adulthood. This runs contrary to the face-expertise argument that face-processing is innate and develops fully in adolescence [21, 22, 26–28]. Instead, our findings suggest that face perception is malleable, and meaningful changes can occur with changes in our environment, and potentially with targeted training. This has important implications for populations that struggle with face processing and emotion perception. Interventions aimed at improving emotion perception impairments have already begun implementing training programs that instruct patients to focus on specific facial features when perceiving and interpreting emotions in others. These interventions have resulted in behavioral improvement in emotion recognition for individuals with traumatic brain injury and those with schizophrenia [70–72]. Though the behavioral changes resulting from these interventions have been widely reported, we know little about the neural mechanisms underlying the effectiveness of these programs. Two recent neuroimaging studies have found promising evidence for increased neural activity after intervention in regions associated with the perception and evaluation of emotions (e.g., the postcentral gyrus, superior occipital lobe, right inferior and superior parietal cortex, and inferior frontal cortex). However, no studies to date have examined short or long-term changes at a microstructural level [73, 74]. Future studies may want to examine neural plasticity more closely in the primary regions associated with both core and extended face processing, such as the OFA, FFA, and amygdala.

Mask exposure has shifted people’s face processing such that people now over utilize certain facial cues. What are the downstream effects of these changes on our social interactions? People use information from eyes and mouth for accurate emotion perception [16, 75, 76]. However, these facial features are not equally important for all emotions—the eye region is particularly important for recognizing sadness and fear, whereas the mouth is more important for happiness and disgust [69, 77]. Thus, masks may be differentially impacting people’s ability to recognize different emotions. Indeed, one recent study that examined the effect of masks on emotion recognition found that participants had difficulty identifying all emotions except fear [78]. Another study that examined emotion perception in young adults in confinement due to COVID found that individuals showed lower accuracy for happiness, and higher accuracy for sadness [79]. These findings mirror what we would expect with the decrease in mouth cue availability—decreased accuracy for happiness–, and an increase in eye cue use—intact recognition of sadness and fearfulness. One limitation of the current study design is the small number of trials per emotion, which prevents investigation into differences in how mask exposure may affect perceptions of unique emotion differently. Future studies may want to examine any differences in the effect of mask exposure on emotion perception across emotions. Additionally, previous research examining the role of mimicry in accurate emotion recognition has also found that limiting a person’s ability to express an emotion themselves, such as by biting down on a pen while making the emotion judgment, impairs their ability to accurately identify the emotion [80, 81]. Though masks may not directly inhibit our ability to express the emotions ourselves, the fact that we cannot fully see the other person’s emotion may interfere with our ability to properly mimic the emotion, and thus may interfere with our ability to accurately identify it. Future studies should more directly explore the effect of facial cue use in emotion perception accuracy during the pandemic.

These shifts in facial cue use should also be considered in a cultural context. For many Americans, this loss of facial information was a significant change in daily life; however, for many other people in the world, interacting with others that have partially occluded faces is more common. For example, prior to the COVID-19 pandemic many people in eastern Asian countries already wore face masks in public during flu seasons or period of high air pollution. Face coverings also play a role in some religious traditions, such as the practice observed by some Muslim women to wear a niqab or burqa when outside of the home. Individuals in these societies may not have been impacted to the same extent by an increase in mask-wearing practices of those around them, or may have already adapted to using other types of cues when perceiving others’ emotions.

Although many studies of emotion perception focus heavily on faces, social information available during real world interactions consists of more than just facial cues. Body language and posture convey a significant amount of information. Indeed, emotions can be recognized correctly just from particular body movements and contextual body cues [82–84]. Vocal cues such as emotional prosody, or “tone of voice”, as well as non-verbal expressions of emotions such as a sigh or laugh, likewise carry significant clues to a speaker’s internal state [85, 86]. One study found that participants were able to perceive emotions from audio clips of naturalistic conversations with a high degree of accuracy [87]. Moreover, the more intensely an emotion was expressed verbally, the higher the accuracy. Thus, masks may not only shift how people use facial cues, but also how people turn to other non-verbal and vocal cues as they attempt to accurately read others’ emotions. Further, as perceivers shift in the cues that they turn to, we might expect to see communicators adapt by shifting in the cues that they produce for receivers. For example, communicators may begin to exaggerate their gestures, their eye movements, or the inflection in their voices in order to compensate for the loss of other facial signals. Future research on this topic may want to continue examining changes to these other types of social signals within the United States, but may also want to look to cultures that already function without access to facial cues to see what types of alternate strategies they may employ.

Social distancing requirements have moved many social interactions online that otherwise would have been conducted in-person. The shift from in-person communication to virtual communication has led to its own shift in the social cues that perceivers have access to, decreasing access to body language and gestures in particular. How has this shift changed the way we read emotional cues? Exploratory measures in our studies examined the effects of virtual interactions, such as talking with people on video calls rather than in person, on cue use (See S1 Appendix for complete analyses). Results from Study 1 revealed that affective cues were less utilized as virtual social interactions increased. One possibility is that as communicators, people don’t feel they need to emote to the same extent while on video calls, thus providing less affective cues to the receiver. Alternatively, there may be some type of “affective flattening” that occurs when people are receiving visual input from others through video, resulting in lower perceptions of affect. These changes might reduce the extent to which arousal is a useful social signal, causing people to focus less on the corresponding facial cues. However, these results were not replicated in Study 2, suggesting that any effect of virtual communication may be small or unreliable, and as such, merits further well-powered investigations in the future.

## Conclusions

The COVID-19 epidemic has led to significant changes in Americans’ daily activities, particularly in the social domain. As people increasingly adopt mask-wearing and social distancing practices, it’s important to understand the impact these changes will have on our social abilities. Our findings run contrary to the previous literature suggesting that face-processing is an innate ability unlikely to be impacted by environmental changes. Instead, changes to incoming perceptual data have shifted the way that people perceive emotions after a mere five months. People are increasingly using visual cues from the faces around them and using arousal cues less the more they’re exposed to others in masks. In light of these findings, we should consider whether our face processing will revert back to a more holistic approach once masks are no longer present in our everyday interactions. The evidence provided here suggests that, as more facial information is available over time, people will again shift to begin using all of the available information.

Though it’s clear the social changes resulting from the COVID-19 pandemic have a significant impact on the way Americans process social information, it’s unclear how long the pandemic and the accompanying behavioral changes will last. But as our studies highlight, humans are adaptive creatures that learn to use the resources available to them and are able to adjust to their environments when necessary.

## Supporting information

S1 Appendix Additional statistical analyses. (DOCX)

S2 Appendix Studies 1 and 2 mask exposure and social interaction measures. (DOCX)

S3 Appendix Pilot Study 1a. (DOCX)
