# Supplementary material for: Mask exposure during COVID-19 changes emotional face processing
Source: PLoS One. 2021 Oct 12;16(10):e0258470. doi: 10.1371/journal.pone.0258470 (PMC8509869; doi:10.1371/journal.pone.0258470)
Supplement: S1 Appendix — (DOCX) [file pone.0258470.s001.docx]

**Additional statistical analyses**

**Study 1**

**Table A. The effect of mask exposure on cue utilization**

| **DV** | ***b*** | ***SE*** | ***t*** | **95% CI** | | ***R^2^*** | ***Adj. R^2^*** |
| --- | --- | --- | --- | --- | --- | --- | --- |
| Eye | .03 | .01 | 2.40 | .006 | .06 | .03 | .02 |
| Mouth | .02 | .01 | 1.53 | −.006 | .05 | 0.01 | .007 |
| Visual | .03 | .01 | 2.16 | .002 | .05 | 0.02 | .02 |
| Arousal | .009 | .01 | .72 | −.02 | .03 | .003 | −.002 |
| Valence | .01 | .01 | .93 | −.02 | .04 | .004 | −.0007 |
| Affective | .02 | .01 | 1.23 | −.01 | .04 | .008 | .003 |

**Table A2. The effect of mask exposure on cue utilization – controlling for age**

| **DV** | ***β*** | ***b*** | ***SE*** | ***t*** | **95% CI** | | ***p*** | ***R^2^*** | ***Adj. R^2^*** |
| --- | --- | --- | --- | --- | --- | --- | --- | --- | --- |
| Eye | .17 | .03 | .01 | 2.43 | .006 | .06 | .02 | .05 | .04 |
| Mouth | .11 | .02 | .01 | 1.55 | -.005 | .04 | .12 | .03 | .02 |
| Visual | .15 | .03 | .01 | 2.12 | .003 | .05 | .03 | .04 | .03 |
| Arousal | .05 | .009 | .01 | .72 | -.02 | .03 | .47 | .005 | -.005 |
| Valence | .07 | .01 | .01 | .93 | -.02 | .04 | .35 | .03 | .02 |
| Affective | .09 | .02 | .01 | 1.23 | -.00003 | .003 | .22 | .03 | .02 |

*The effects of social interactions*

Here we tested whether social interaction predicted participants’ use of different cues to determine facial similarity. We conducted separate simple linear regressions to predict each cue (global visual, eye, mouth, affective, valence, arousal) as a function of social interaction. Results showed that social interaction alone did not have an impact on any of the cue types (eye: *β* = 0.02, *p* = 0.80; mouth: *β* = -0.003, *p* = 0.97; global visual: *β* = 0.02, *p* = 0.77, affective: *β* = -0.02, *p* = 0.78; valence: *β* = -0.03, *p* = 0.64, arousal: *β* = 0.05, *p* = 0.44) (See Table B for full model statistics).

**Table B.** **The effect of social Interaction on cue utilization**

| **DV** | ***b*** | ***SE*** | ***t*** | **95% CI** | | ***R^2^*** | ***Adj. R^2^*** |
| --- | --- | --- | --- | --- | --- | --- | --- |
| Eye | 0.003 | .01 | .26 | −0.02 | 0.03 | 0.0003 | −0.005 |
| Mouth | −.0005 | .01 | −.04 | −.02 | .02 | 9.09e-06 | −0.005 |
| Visual | .004 | .01 | .29 | −.02 | .03 | .0004 | −0.005 |
| Arousal | .009 | .01 | .77 | −.01 | .03 | .003 | −.002 |
| Valence | −.007 | .01 | −.47 | −.03 | .02 | .001 | −.004 |
| Affective | −.004 | .01 | −.29 | −.03 | .02 | .0004 | −0.005 |

**Table B2. The effect of social interaction on cue utilization – controlling for age**

| **DV** | ***β*** | ***b*** | ***SE*** | ***t*** | **95% CI** | | ***p*** | ***R^2^*** | ***Adj. R^2^*** |
| --- | --- | --- | --- | --- | --- | --- | --- | --- | --- |
| Eye | .007 | .001 | .01 | .09 | -.02 | .03 | .93 | .02 | .01 |
| Mouth | -.01 | -.002 | .01 | -.20 | -.03 | .02 | .84 | .02 | .01 |
| Visual | .01 | .002 | .01 | .15 | -.02 | .03 | .88 | .02 | .007 |
| Arousal | .05 | .008 | .01 | .72 | -.04 | .03 | .47 | .005 | -.005 |
| Valence | -.02 | -.004 | .01 | -.30 | -.03 | .02 | .76 | .02 | .01 |
| Affective | -.01 | -.002 | .01 | -.14 | -.03 | .02 | .89 | .02 | .009 |

**Table C. The effect of Mask exposure on cue utilization moderated by social interaction**

| **Outcomes** | **Eye cue** | | | | | **Mouth cue** | | | | | | |
| --- | --- | --- | --- | --- | --- | --- | --- | --- | --- | --- | --- | --- |
| Predictors | ***b*** | **SE** | ***t*** | **95% CI** | | ***b*** | **SE** | | ***t*** | **95% CI** | | |
| Social interaction | −.003 | .01 | −.23 | −.03 | .02 | −.004 | .01 | | −.35 | −.03 | | .02 |
| Mask exposure | .03 | .01 | 2.38 | .006 | .06 | .02 | .01 | | 1.57 | −.005 | | .05 |
| Social interaction x Mask exposure | −.004 | .02 | −.19 | −.05 | .04 | .003 | .02 | | .16 | −.04 | | .04 |
| R^2^ | .03 | | | | | .01 | | | | | | |
| Adj. R^2^ | .01 | | | | | −.003 | | | | | | |
| **Outcomes** | **Arousal cue** | | | | | **Valence cue** | | | | | | |
| Predictors | ***b*** | **SE** | ***t*** | **95% CI** | | ***b*** | | **SE** | ***t*** | | **95% CI** | |
| Social interaction | .008 | .01 | .70 | −.02 | .03 | −.01 | | .01 | −.73 | | −.04 | .02 |
| Mask exposure | .007 | .01 | .59 | −.02 | .03 | .02 | | .02 | 1.04 | | −.01 | .05 |
| Social interaction x Mask exposure | .006 | .02 | .33 | −.03 | .04 | −.002 | | .02 | −.09 | | −.05 | .04 |
| R^2^ | .006 | | | | | .007 | | | | | | |
| Adj. R^2^ | −.01 | | | | | −.008 | | | | | | |
| **Outcomes** | **Global visual cue** | | | | | **Affective cue** | | | | | | |
| Predictors | ***b*** | **SE** | ***t*** | **95% CI** | | ***b*** | | **SE** | ***t*** | | **95% CI** | |
| Social interaction | −.002 | .01 | −.15 | −.03 | .02 | −.008 | | .01 | −.56 | | −.03 | .02 |
| Mask exposure | .03 | .01 | 2.12 | .002 | .05 | .02 | | .01 | 1.31 | | −.01 | .05 |
| Social interaction x Mask exposure | −.009 | .02 | −.47 | −.05 | .03 | −.003 | | .02 | −.14 | | −.05 | .04 |
| R^2^ | .02 | | | | | .009 | | | | | | |
| Adj. R^2^ | .009 | | | | | -.006 | | | | | | |

**Table C2. The effect of Mask exposure on cue utilization moderated by social interaction – controlling for age**

| **Outcomes** | **Eye cue** | | | | | | |
| --- | --- | --- | --- | --- | --- | --- | --- |
| Predictors | ***β*** | ***b*** | ***SE*** | ***t*** | **95% CI** | | ***p*** |
| Social interaction | -.03 | -.005 | .01 | -.41 | -.03 | .02 | .68 |
| Mask exposure | .17 | .03 | .01 | 2.44 | .007 | .06 | .02 |
| Age | -.16 | -.002 | .0008 | -2.24 | -.003 | -.0002 | .03 |
| Social interaction x Mask exposure | -.01 | -.004 | .02 | -.18 | -.05 | .04 | .86 |
| R^2^ | .05 | | | | | | |
| Adj. R^2^ | .03 | | | | | | |
| **Outcomes** | **Mouth cue** | | | | | | |
| Predictors | ***β*** | ***b*** | ***SE*** | ***t*** | **95% CI** | | ***p*** |
| Social interaction | -.04 | -.006 | .01 | -.52 | -.03 | .02 | .60 |
| Mask exposure | .12 | .02 | .01 | 1.62 | -.005 | .05 | .11 |
| Social interaction x Mask exposure | .01 | .003 | .02 | .18 | -.04 | .04 | .86 |
| Age | -.15 | -.002 | .0007 | -2.10 | -.003 | -9.3e-05 | .04 |
| R^2^ | .03 | | | | | | |
| Adj. R^2^ | .01 | | | | | | |
| **Outcomes** | **Global visual cue** | | | | | | |
| Predictors | ***β*** | ***b*** | ***SE*** | ***t*** | **95% CI** | | ***p*** |
| Social interaction | -.02 | -.004 | .01 | -.30 | -.03 | .02 | .76 |
| Mask exposure | .16 | .03 | .01 | 2.17 | .003 | .05 | .03 |
| Social interaction x Mask exposure | -.03 | -.009 | .02 | -.46 | -.05 | .03 | .65 |
| Age | -.13 | -.001 | .0007 | -1.87 | -.003 | .00008 | .06 |
| R^2^ | .04 | | | | | | |
| Adj. R^2^ | .02 | | | | | | |
| **Outcomes** | **Arousal cue** | | | | | | |
| Predictors | ***β*** | ***b*** | ***SE*** | ***t*** | **95% CI** | | ***p*** |
| Social interaction | .05 | .008 | .01 | .65 | -.02 | .03 | .52 |
| Mask exposure | .04 | .007 | .01 | .60 | -.02 | .03 | .55 |
| Social interaction x Mask exposure | .02 | .006 | .02 | .33 | -.03 | .04 | .74 |
| Age | -.04 | -.0004 | .0007 | -.59 | -.002 | .0009 | .55 |
| R^2^ | .007 | | | | | | |
| Adj. R^2^ | -.01 | | | | | | |
| **Outcomes** | **Valence cue** | | | | | | |
| Predictors | ***β*** | ***b*** | ***SE*** | ***t*** | **95% CI** | | ***p*** |
| Social interaction | -.04 | -.00 | .01 | -.56 | -.04 | .02 | .57 |
| Mask exposure | .07 | .02 | .02 | 1.01 | -.01 | .04 | .31 |
| Social interaction x Mask exposure | -.008 | -.002 | .02 | -.11 | -.05 | .04 | .91 |
| Age | .15 | .002 | .0008 | 2.12 | .0001 | .003 | .03 |
| R^2^ | .03 | | | | | | |
| Adj. R^2^ | .01 | | | | | | |
| **Outcomes** | **Affective cue** | | | | | | |
| Predictors | ***β*** | ***b*** | ***SE*** | ***t*** | **95% CI** | | ***p*** |
| Social interaction | -.03 | -.006 | .01 | -.41 | -.03 | .02 | .68 |
| Mask exposure | .09 | .02 | .01 | 1.23 | -.01 | .05 | .20 |
| Social interaction x Mask exposure | -.01 | -.003 | .02 | -.16 | -.05 | .03 | .88 |
| Age | .13 | .001 | .0008 | 1.89 | -.00006 | .003 | .06 |
| R^2^ | .03 | | | | | | |
| Adj. R^2^ | .007 | | | | | | |

*The effects of virtual social interactions*

Here we examined the effects of virtual social interactions, that is interactions with people and faces that are through a technological outlet rather than in person, on cue utilization. We conducted separate simple linear regressions to predict each cue (global visual, eye, mouth, affective, valence, arousal) as a function of virtual social interactions. Results revealed a significant effect of virtual social interactions on affective (*β* = -0.20, *p* = 0.004) and valence cues (*β* = -0.24, *p* < 0.001), such that affective and valence cues were less utilized as virtual social interactions increased. Three was no effect of virtual social interaction on eye (*β* = -0.008, *p* = 0.91), mouth (*β* = -0.09, *p* = 0.22), global visual (*β* = -0.03, *p* = 0.65) or arousal (*β* = 0.02, *p* = 0.74) cues (See Table D for full model statistics). These results suggest that virtual social interactions, such as talking with people on video calls rather than in person, may be reducing our ability to pick up on affective cues in facial expressions.

**Table D. The effect of virtual social interactions on cue utilization**

| **DV** | ***b*** | ***SE*** | ***t*** | **95% CI** | | ***R^2^*** | ***Adj. R^2^*** |
| --- | --- | --- | --- | --- | --- | --- | --- |
| Eye | −.001 | .01 | −.12 | −.03 | .02 | 6.86e-05 | −.005 |
| Mouth | −.01 | .01 | −1.23 | −.04 | .008 | .008 | .003 |
| Visual | −.005 | .01 | −.45 | −.03 | .02 | .001 | −.004 |
| Arousal | .004 | .01 | .34 | −.02 | .02 | .0006 | −.004 |
| Valence | −.05 | .01 | −3.54 | −.07 | −.02 | .06 | .05 |
| Affective | −.04 | .01 | −2.93 | −.06 | −.01 | .04 | .004 |

*Controlling for all cue types*

People likely use multiple types of information, in parallel, to make their emotional face judgments. Our initial analyses treated each type of cue independently. To address this issue, we conducted additional analyses where we incorporate multiple cue types into the same analysis. Specifically, we performed two new multiple regressions predicting participant’s similarity ratings that control for all cue types: First, we conducted a multiple regression predicting participant’s similarity ratings from four ground-truth similarity scores: valence, arousal, eye region, and mouth region cues. We used the coefficients from this analysis as the new cue use scores for each cue type (valence, arousal, eye, mouth). Second, we conducted a multiple regression predicting participant’s similarity ratings from two ground-truth similarity scores: global visual and affective cues. We used the resulting coefficients from this model as the new global visual and affective cue use scores.

We then conducted our primary analyses again, using these new cue use scores. We first tested whether mask exposure predicted participants’ use of different facial cues in their ratings of emotion similarity. Results revealed that mask exposure significantly predicted an increase in eye cues (*β* = .15, b = .05, SE = .02, t = 2.19, 95% CI = [.005, .09], *p* = .03), but not mouth cues (*β* = -.07, b = -.02, SE = .02, t = -1.0, 95% CI = [-.06,.02], *p* = .31). There was a non-significant trend for an increase in global visual cues (*β* = 0.14, b = .03, SE = .01, t = 1.95, 95% CI = [-.0003, .05], *p* = 0.05). Mask exposure did not have a significant impact on use of affective (*β* = .06, b = .01, SE = .01, t = .85, 95% CI = [-.02,.04], *p* = .40), valence (*β* = .07, b = .01, SE = .02, t = .95, 95% CI = [-.02, .04], *p* = .35), or arousal cues (*β* = .03, b = .005, SE = .01, t = .44, 95% CI = [-.02, .03], *p* = .66). Next, we tested whether exposure to masks affected cue use differently depending on the amount of social interaction people engaged in. We conducted six additional regressions with each cue (global visual, eye, mouth, affective, valence, arousal) as the DV and the interaction of mask exposure and social interaction as the predictor. Results showed no significant effect of the interaction between mask exposure and social interaction on any of the cue types [eye: (*β* = -.04, b = -.02, SE = .03 t = -.56, 95% CI = [-.08, .05], *p* .58); mouth: (*β* = .04, b = .02, SE = .03, t = .60, 95% CI = [-.04, .08], *p* = .55); global visual: (*β* =-.03, b = -.009, SE = .02, t = -.45, 95% CI = [-.05, .03], *p* = .65), affective: (*β* = -.004, b = -.001, SE = .02, t = -.05, 95% CI = [-.04, .04], *p* =.96); valence: (*β* = -.02, b = -.005, SE = .02, t = -.23, 95% CI = [-.05, .04], *p* = .82), arousal(*β* = .03, b = .007, SE = .02, t = .38, 95% CI = [-.03, .04], *p* = .71)].

**Study 2**

**Table E. The effect of mask exposure on the difference in cue utilization between time 1 and time 2**

| **DV** | ***b*** | ***SE*** | ***t*** | **95% CI** | | ***R^2^*** | ***Adj. R^2^*** |
| --- | --- | --- | --- | --- | --- | --- | --- |
| Eye | −.01 | .02 | −.66 | −.06 | .03 | .003 | −.004 |
| Mouth | .001 | .02 | .06 | −.04 | .04 | 2.25e-05 | −.007 |
| Visual | −.002 | .02 | −.10 | −.04 | .04 | 7.11e-05 | −.007 |
| Arousal | −.03 | .02 | −1.33 | −.07 | .01 | .01 | .005 |
| Valence | −.001 | .02 | −.05 | −.05 | .05 | 1.60e-05 | −.007 |
| Affective | −.005 | .02 | −.23 | −.05 | .04 | .0004 | −.007 |

**Table E2. The effect of mask exposure on the difference in cue utilization between time 1 and time 2 – controlling for age**

| **DV** | ***β*** | ***b*** | ***SE*** | ***t*** | **95% CI** | | ***p*** | ***R^2^*** | ***Adj. R^2^*** |
| --- | --- | --- | --- | --- | --- | --- | --- | --- | --- |
| Eye | -.06 | -.02 | .03 | -.74 | -.06 | .03 | .46 | .01 | -.004 |
| Mouth | -.005 | -.001 | .02 | -.06 | -.04 | .04 | .96 | .01 | -.003 |
| Visual | -.02 | -.004 | .02 | -.21 | -.05 | .04 | .83 | .01 | -.003 |
| Arousal | -.11 | -.03 | .02 | -1.36 | -.07 | .01 | .18 | .01 | -.0008 |
| Valence | -.02 | -.005 | .02 | -.20 | -.05 | .04 | .84 | .02 | .007 |
| Affective | -.03 | -.008 | .02 | -.33 | -.06 | .04 | .74 | .01 | -.004 |

*The effects of social interactions on difference in cue utilization*

Here we tested whether amount of social interaction could account for the difference in cue use between time 1 and time 2. To do so, we computed difference scores for each cue type, subtracting time 1 scores from time 2 scores, such that positive scores reflect higher use at time 2 and negative scores reflect higher use at time 1. We conducted separate simple linear regressions with each difference in cue use as the DV predicted by social interaction. Results showed that the difference in cue use between time 1 and time 2 could not be explained by social interaction alone (eye: *β* = 0.02, *p* = 0.82; mouth: *β* = 0.04, *p* = 0.59; global visual *β* = 0.04, *p* = 0.61; arousal: *β* = -0.08, *p* = 0.32; valence: *β* = -0.04, *p* = 0.66; affective: *β* = -0.05, *p* = 0.57; Table F for full model statistics).

**Table F. The effect of social interaction on the difference in cue utilization between time 1 and time 2**

| **DV** | ***b*** | ***SE*** | ***t*** | **95% CI** | | ***R^2^*** | **Adj*.R^2^*** |
| --- | --- | --- | --- | --- | --- | --- | --- |
| Eye | .004 | .02 | .23 | −.03 | .04 | .0004 | −.007 |
| Mouth | .009 | .02 | .53 | −.02 | .04 | .002 | −.005 |
| Visual | .009 | .02 | .51 | −.03 | .04 | .002 | −.005 |
| Arousal | −.02 | .02 | −1.01 | −.05 | .02 | .007 | .00009 |
| Valence | −.009 | .02 | −.44 | −.05 | .03 | .001 | −.006 |
| Affective | −.01 | .02 | −0.57 | −.05 | .03 | .002 | −.005 |

**Table F2. The effect of social interaction on the difference in cue utilization between time 1 and time 2 – controlling for age**

| **DV** | ***β*** | ***b*** | ***SE*** | ***t*** | **95% CI** | | ***p*** | ***R^2^*** | ***Adj. R^2^*** |
| --- | --- | --- | --- | --- | --- | --- | --- | --- | --- |
| Eye | .01 | .002 | .02 | .13 | -.03 | .04 | .89 | .007 | -.007 |
| Mouth | .03 | .007 | .02 | .41 | -.03 | .04 | .68 | .01 | -.002 |
| Visual | .03 | .007 | .02 | .39 | -.03 | .04 | .70 | .01 | -.002 |
| Arousal | -.09 | -.02 | .02 | -1.03 | -.05 | .02 | .31 | .008 | -.006 |
| Valence | -.05 | -.01 | .02 | -.62 | -.05 | .03 | .54 | .02 | .009 |
| Affective | -.06 | -.01 | .02 | -.69 | -.05 | .03 | .49 | .01 | -.001 |

**Table G. The effect of Mask exposure on cue utilization difference between time 1 and time 2 by social interaction**

| **Outcomes** | **Eye difference** | | | | | | | **Mouth difference** | | | | |
| --- | --- | --- | --- | --- | --- | --- | --- | --- | --- | --- | --- | --- |
| Predictors | ***b*** | **SE** | | | ***t*** | **95% CI** | | ***b*** | **SE** | ***t*** | **95% CI** | |
| Social interaction | .004 | | .02 | .20 | | −.03 | .04 | .007 | .02 | .41 | −.03 | .04 |
| Mask exposure | −.009 | | .02 | −.38 | | −.05 | .04 | .004 | .02 | .17 | −.04 | .05 |
| Social interaction x Mask exposure | .07 | | .03 | 2.30 | | .01 | .13 | .05 | .03 | 1.60 | −.01 | .11 |
| R^2^ | .04 | | | | | | | .02 | | | | |
| Adj. R^2^ | .02 | | | | | | | −.001 | | | | |
| **Outcomes** | **Arousal difference** | | | | | | | **Valence difference** | | | | |
| Predictors | ***b*** | | **SE** | ***t*** | | **95% CI** | | ***b*** | **SE** | ***t*** | **95% CI** | |
| Social interaction | −.02 | | .02 | −.72 | | −.05 | .02 | −.009 | .02 | −.43 | −.05 | .03 |
| Mask exposure | −.02 | | .02 | −1.12 | | −.07 | .02 | .0009 | .03 | .03 | −.05 | .05 |
| Social interaction x Mask exposure | −.0007 | | .03 | −.02 | | −.06 | .06 | −.004 | .04 | −.02 | −.07 | .07 |
| R^2^ | .02 | | | | | | | .001 | | | | |
| Adj. R^2^ | −.005 | | | | | | | −.02 | | | | |
| **Outcomes** | **Visual difference** | | | | | | | **Visual difference** | | | | |
| Predictors | ***b*** | **SE** | | | ***t*** | **95% CI** | | ***b*** | **SE** | ***t*** | **95%CI** | |
| Social interaction | .006 | | .02 | .37 | | −.03 | .04 | −.01 | .02 | −.52 | −.05 | .03 |
| Mask exposure | .003 | | .02 | .12 | | −.04 | .04 | −.002 | .03 | −.11 | −.05 | .05 |
| Social interaction x Mask exposure | .07 | | .03 | 2.30 | | .01 | .13 | −.002 | .03 | −.07 | −.07 | .07 |
| R^2^ | .04 | | | | | | | .002 | | | | |
| Adj. R^2^ | .02 | | | | | | | −.02 | | | | |

**Table G2. The effect of Mask exposure on cue utilization difference between time 1 and time 2 by social interaction – controlling for age**

| **Outcomes** | **Eye cue** | | | | | | |
| --- | --- | --- | --- | --- | --- | --- | --- |
| Predictors | ***β*** | ***b*** | ***SE*** | ***t*** | **95% CI** | | ***p*** |
| Social interaction | .01 | .002 | .02 | .12 | -.03 | .04 | .90 |
| Mask exposure | -.04 | -.01 | .02 | -.45 | -.05 | .03 | .65 |
| Social interaction x Mask exposure | .19 | .07 | .03 | 2.29 | .01 | .13 | .02 |
| Age | -.08 | -.001 | .001 | -.99 | -.003 | .001 | .33 |
| R^2^ | .05 | | | | | | |
| Adj. R^2^ | .02 | | | | | | |
| **Outcomes** | **Mouth cue** | | | | | | |
| Predictors | ***β*** | ***b*** | ***SE*** | ***t*** | **95% CI** | | ***p*** |
| Social interaction | .03 | .005 | .02 | .31 | -.03 | .04 | .76 |
| Mask exposure | .008 | .002 | .02 | .09 | -.04 | .04 | .93 |
| Social interaction x Mask exposure | .13 | .05 | .03 | 1.58 | -.01 | .10 | .12 |
| Age | -.10 | -.001 | .001 | -1.21 | -.003 | .0008 | .23 |
| R^2^ | .03 | | | | | | |
| Adj. R^2^ | .002 | | | | | | |
| **Outcomes** | **Global visual cue** | | | | | | |
| Predictors | ***β*** | ***b*** | ***SE*** | ***t*** | **95% CI** | | ***p*** |
| Social interaction | .02 | .005 | .02 | .27 | -.03 | .04 | .79 |
| Mask exposure | .003 | .0008 | .02 | .04 | -.04 | .04 | .97 |
| Social interaction x Mask exposure | .19 | .07 | .03 | 2.29 | .009 | .13 | .02 |
| Age | -.10 | -.001 | .001 | -1.20 | -.003 | .0008 | .23 |
| R^2^ | .05 | | | | | | |
| Adj. R^2^ | .02 | | | | | | |
| **Outcomes** | **Arousal cue** | | | | | | |
| Predictors | ***β*** | ***b*** | ***SE*** | ***t*** | **95% CI** | | ***p*** |
| Social interaction | -.06 | -.01 | .02 | -.75 | -.05 | .02 | .46 |
| Mask exposure | -.10 | -02 | .02 | -1.15 | -.07 | .02 | .25 |
| Social interaction x Mask exposure | -.002 | -.0008 | .03 | -.03 | -.06 | .06 | .98 |
| Age | -.03 | -.0004 | .001 | -.41 | -.002 | .002 | .69 |
| R^2^ | .02 | | | | | | |
| Adj. R^2^ | -.01 | | | | | | |
| **Outcomes** | **Valence cue** | | | | | | |
| Predictors | ***β*** | ***b*** | ***SE*** | ***t*** | **95% CI** | | ***p*** |
| Social interaction |  | -.01 | .02 | -.57 |  |  | .57 |
| Mask exposure |  | -.002 | .03 | -.09 |  |  | .93 |
| Social interaction x Mask exposure |  | -.005 | .04 | -.14 |  |  | .89 |
| Age |  | -.002 | .001 | -1.78 |  |  | .08 |
| R^2^ | .02 | | | | | | |
| Adj. R^2^ | -.004 | | | | | | |
| **Outcomes** | **Affective cue** | | | | | | |
| Predictors | ***β*** | ***b*** | ***SE*** | ***t*** | **95% CI** | | ***p*** |
| Social interaction | -.05 | -.01 | .02 | -.62 | -.05 | .03 | .54 |
| Mask exposure | -.02 | -.005 | .03 | -.19 | -.05 | .04 | .85 |
| Social interaction x Mask exposure | -.007 | -.003 | .03 | -.09 | -.07 | .07 | .93 |
| Age | -.10 | -.001 | .001 | -1.21 | -.004 | .0009 | .23 |
| R^2^ | .01 | | | | | | |
| Adj. R^2^ | -.02 | | | | | | |

**Table H. Conditional effects of Mask Exposure on cue utilization difference between time 1 and time 2**

|  | **Eye difference** | | | | **Visual difference** | | | |
| --- | --- | --- | --- | --- | --- | --- | --- | --- |
| Social Interaction | ***b*** | ***p*** | **95% CI** | | ***b*** | ***p*** | **95% CI** | |
| Min (-1.30) | −.10 | .02 | −.19 | −.02 | −.09 | .04 | −.17 | −.005 |
| Mean (0) | −.01 | .70 | −.05 | .04 | .003 | .90 | −.04 | .05 |
| Max (1.95) | .13 | .06 | −.00 | .27 | .14 | .04 | .01 | .27 |

*The effects of virtual social interactions on difference in cue utilization*

We conducted exploratory analyses to examine the effects of virtual social interactions on cue utilization. We conducted separate simple linear regressions to predict each cue difference (global visual, eye, mouth, affective, valence, arousal) as a function of virtual social interactions. Results revealed that virtual social interactions did not significantly account for the difference in any cue utilization (eye: *β* = -0.15, *p* = 0.07; mouth: *β* = -0.10, *p* = 0.24; global visual: *β* = -0.09, *p* = 0.29; arousal: *β* = 0.04, *p* = 0.63; valence: *β* = -0.02, *p* = 0.80; affective: *β* = 0.004, *p* = 0.96) (See Table I for full model statistics).

**Table I. The effect of virtual social interaction on the difference in cue utilization between time 1 and time 2.**

| **DV** | ***b*** | **SE** | ***t*** | **95% CI** | | **R2** | **Adj. R2** |
| --- | --- | --- | --- | --- | --- | --- | --- |
| Eye | −.03 | .02 | −1.8 | −.07 | .003 | .02 | .02 |
| Mouth | −.02 | .02 | −1.18 | −.06 | .01 | .01 | .003 |
| Visual | −.02 | .02 | −1.06 | −.06 | .02 | .008 | .0008 |
| Arousal | .009 | .02 | .48 | −.03 | .04 | .002 | −.005 |
| Valence | −.005 | .02 | −.25 | −.05 | .04 | .0004 | −.007 |
| Affective | .001 | .02 | .05 | −.04 | .04 | 1.98E-05 | −.007 |

*Controlling for all cue types*

As in Study 1, we performed two new multiple regressions predicting participant’s similarity ratings that control for all cue types: 1) a multiple regression predicting participant’s similarity ratings from four ground-truth similarity scores: valence, arousal, eye region, and mouth region cues, and 2) a multiple regression predicting participant’s similarity ratings from two ground-truth similarity scores: global visual and affective cues. We used the resulting coefficients from these models as the new cue use scores for each cue type (valence, arousal, eye, mouth, global visual and affective). We then conducted our primary analyses again, using these new cue use scores.

First, we examined whether cumulative mask exposure over the course of five months increased the extent to which people rely on visual and affective face cues when making emotion perception judgments. Results revealed that people used global visual cues to a greater extent at time 2 compared to time 1 (*t*(145) = 4.27, *p* < 0.001, *d* = 0.35, CI:[.03, .08]). However, we did not observe a specific increase in the eye (*t*(145) = 1.21, *p* = .23, *d* = .1, CI: [-.02, .07]) or mouth(*t*(145) = 1.11, *p* =.27, *d* = 0.09, CI: [-.02, .06]) regions. In addition, we found evidence that people used affective cues to a lesser extent at time 2 compared to time 1 (*t*(145) = -2.5, *p* =.01, *d* = 0.21. CI: [-.06, -.007]), specifically arousal cues (*t*(145) = -5.07, *p* < 0.001, *d* = 0.42, CI: [-.08, -.04]). There was no significant differences between timepoints for valence cues (*t*(145) = -.02, *p* = 84, *d* = .02, CI: [-.03, .03]).

Next, we tested whether exposure to masks between time 1 and time 2 could account for the difference in cue use between time 1 and time 2. To test this, we first computed difference scores for each cue type, subtracting time 1 scores from time 2 scores, such that positive scores reflect higher cue use at time 2 and negative scores reflect higher cue use at time 1. We then conducted separate simple linear regressions with each difference in cue use as the DV predicted by mask exposure. Results showed that the difference in cue use between time 1 and time 2 could not be explained by mask exposure alone [eye: (*β* = -.08, b = -.04, SE = .04, t = -.97, 95% CI = [-.11, .04], *p =* .33); mouth: (*β* = .07, b = .03, SE = .04, t = .83, 95% CI = [-.04, .10], *p* = .41); visual: (*β* = -.006, b = -.002, SE = .02, t = -.07, 95% CI = -.04, .04], *p* = .94); affective(*β* = -.02, b = -.006, SE = .02, t = -.25, 95% CI = [-.05, .04], *p* = .81)]; arousal: (*β* = -.10, b = -.03, SE = .02, t = -1.30, 95% CI = [-.07, .01], *p* = .20); valence (*β* = -.01, b = -.003, SE = .03, t = -.14, 95% CI =[ -.05, .05], *p* = .89)].

Finally, we examined whether mask exposure would impact participants differently depending on the amount of social interaction they engaged in by regressing the interaction between mask exposure and social interaction onto the difference in cue use, separately for each cue type. The interactions were probed using the *sim_slope()* function of the interactions package (v1.1.3) by testing the conditional effects of mask exposure at 3 levels of social interaction: at the minimum value, the mean, and the maximum value.

Results revealed a significant interaction for global visual cues, such that the effect of mask exposure changed at different levels of social interaction (*β* = .20, b = .07, SE = .03, t = 2.42, 95% CI = [.01, .13], *p* = .02). Analysis of the simple effects showed that for people with the highest levels of social interaction, use of global visual cues increased as mask exposure increased (*b* = .14, 95% CI = [.02, .27], t = 2.21, *p* = .03); for people with the least social interaction, use of global visual cues decreased as mask exposure increased (*b* = -.09, 95% CI = [-0.17, -.009], t = -2.2, *p* = .03). There was no effect of mask exposure on people with average social interaction for global visual cues (*b* = .003, 95% CI = [-.04, .05], t = .12, *p* = .91).

There were no interaction effects between mask exposure and social interaction for the specific facial regions: eye cues (*β* = .13, b = .08, SE = .05, t = 1.6, 95% CI = [-.02, .19], *p* = .11) or mouth cues (*β* = -.03, b = -.02, SE = .05, t = -.32, 95% CI =[-.12, .09], *p* = .75). There were also no significant interaction effects for affective cues (*β* = -.05, b = -.02, SE = .04, t = -.56, 95% CI =[-.09, .05], *p* = .58), arousal cues (*β* = .15, b = -.005, SE = .03, t = -.16, 95% CI = [-.06, .05], *p* = .87), nor valence cues (*β* = -.02, b = -.007, SE = .04, t = -.19, 95% CI = [-.08, .07], *p* = .85).
