# Supplementary material for: Mask exposure during COVID-19 changes emotional face processing
Source: PLoS One. 2021 Oct 12;16(10):e0258470. doi: 10.1371/journal.pone.0258470 (PMC8509869; doi:10.1371/journal.pone.0258470)
Supplement: S2 Appendix — (DOCX) [file pone.0258470.s002.docx]

**Studies 1 and 2 mask exposure and social interaction measures**

Mask Exposure

*Continuous Scale from 0-100*

1. What percentage of time do you personally wear a mask while engaging in activities outside of the home?

|  | 0 | 25 | 50 | 75 | 100 |
| --- | --- | --- | --- | --- | --- |


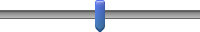


1. What percentage of people in your household wear masks while engaging in activities outside of the home?

|  | 0 | 25 | 50 | 75 | 100 |
| --- | --- | --- | --- | --- | --- |


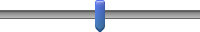


1. What percentage of people in your local community wear masks while engaging in activities outside of the home?

|  | 0 | 25 | 50 | 75 | 100 |
| --- | --- | --- | --- | --- | --- |


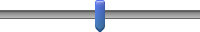


1. Please report the percentage of people wearing masks while you were engaged in the following activities over the *last 24-hours*:

4a. Around other people in the same physical space, but not interacting with

them: (e.g. in a coffeeshop, library, office building, bus, gym, waiting room, store,

etcetera):

Percent of people: ______

4b. Interacting with other face-to-face:

Percent of people: _______

1. Please report the percentage of people wearing masks while you were engaged in the following activities over *the last month*:

5a. Around other people in the same physical space, but not interacting with

them: (e.g. in a coffeeshop, library, office building, bus, gym, waiting room, store,

etcetera):

Percent of people: ______

5b. Interacting with other face-to-face:

Percent of people: _______

Social Interaction

1. Please report how many total hours you spent engaged in the following activities over the *last 24-hours*:

1a. Around other people in the same physical space, but not interacting with

them (e.g. in a coffeeshop, library, office building, bus, gym, waiting room, store,

etcetera)

Number of hours: _____

1b. Interacting with others face-to-face

Number of hours: _____

1c. Talking to one or more people on video calls such as Skype or FaceTime

Number of hours: _____

1d. Watching TV shows or movies

Number of hours: _____

1. Please report how many total people you engaged with in the following activities over the *last 24-hours*:

2a. Around other people in the same physical space, but not interacting with

them: (e.g. in a coffeeshop, library, office building, bus, gym, waiting room, store,

etcetera)

Number of people: _____

2b. Interacting with others face-to-face

Number of people: _____

2c. Talking to one or more people on video calls such as Skype or FaceTime

Number of hours: _____

1. Please report how many total hours you spent engaged in the following activities over the *last month*:

3a. Around other people in the same physical space, but not interacting with

Them (e.g. in a coffeeshop, library, office building, bus, gym, waiting room, store,

etcetera)

Number of hours: _____

3b. Interacting with others face-to-face

Number of hours: _____

3c. Talking to one or more people on video calls such as Skype or FaceTime

Number of hours: _____

3d. Watching TV shows or movies

Number of hours: _____

1. Please report how many total people you engaged with in the following activities over the *last month*:

4a. Around other people in the same physical space, but not interacting with

them: (e.g. in a coffeeshop, library, office building, bus, gym, waiting room, store,

etcetera)

Number of people: _____

4b. Interacting with others face-to-face

Number of people: _____

4c. Talking to one or more people on video calls such as Skype or FaceTime

Number of hours: _____
