# Supplementary material for: Mask exposure during COVID-19 changes emotional face processing
Source: PLoS One. 2021 Oct 12;16(10):e0258470. doi: 10.1371/journal.pone.0258470 (PMC8509869; doi:10.1371/journal.pone.0258470)
Supplement: S3 Appendix — (DOCX) [file pone.0258470.s003.docx]

**Pilot Study 1a**

A pilot study was conducted to validate our measures of mask exposure and social interaction.

**Participants**

Participants were recruited through Amazon’s Mechanical Turk to complete a 20-minute study for $2.00. Study 1a was an initial pilot study, and the target sample size set to 50 to detect a small effect size. Participation was restricted to mTurk workers in the United States with > 95% approval ratings. Participants were excluded if they reported that an English comprehension less than “Good” (N = 0), completed less than half of the questions of interest (N = 8), or reported social interaction above the 75^th^ or below the 25^th^ percentile by a factor of 1.5 times the interquartile range (N = 5), for a final sample size of 37.

**Methods**

**Mask Exposure**

*Continuous scale from 0-100*

1. What percentage of time do you personally wear a mask while engaging in activities outside of the home?
2. What percentage of people in your household wear masks while engaging in activities outside of the home?
3. What percentage of people in your local community wear masks while engaging in activities outside of the home?

*Short text entry (Percent of people)*

1. Please report the percentage of people wearing masks while you were engaged in the following activities over the *last 24-hours*:

4a. Around other people in the same physical space, but not interacting with

them: (e.g. in a coffeeshop, library, office building, bus, gym, waiting room, store,

etcetera):

4b. Interacting with others face-to-face:

4c. Talking to one or more people on video calls such as Skype or FaceTime

4d. Watching TV shows or movies

1. Please report the percentage of people wearing masks while you were engaged in the following activities over *the last month*:

5a. Around other people in the same physical space, but not interacting with

them: (e.g. in a coffeeshop, library, office building, bus, gym, waiting room, store,

etcetera)

5b. Interacting with others face-to-face

5c. Talking to one or more people on video calls such as Skype or FaceTime

5d. Watching TV shows or movies

**Social Interaction**

*Short Text Entry (Number of hours)*

1. Please report how many total hours you spent engaged in the following activities over the *last 24-hours*:

1a. Around other people in the same physical space, but not interacting with

Them (e.g. in a coffeeshop, library, office building, bus, gym, waiting room, store,

etcetera)

1b. Interacting with others face-to-face

1c. Talking to one or more people on video calls such as Skype or FaceTime

1d. Watching TV shows or movies

*Short Text Entry (Number of People)*

1. Please report how many total people you engaged with in the following activities over the *last 24-hours*:

2a. Around other people in the same physical space, but not interacting with

them: (e.g. in a coffeeshop, library, office building, bus, gym, waiting room, store,

etcetera)

2b. Interacting with others face-to-face

2c. Talking to one or more people on video calls such as Skype or FaceTime

*Short Text Entry (Number of hours)*

1. Please report how many total hours you spent engaged in the following activities over the *last month*:

3a. Around other people in the same physical space, but not interacting with

Them (e.g. in a coffeeshop, library, office building, bus, gym, waiting room, store,

etcetera)

3b. Interacting with others face-to-face

3c. Talking to one or more people on video calls such as Skype or FaceTime

3d. Watching TV shows or movies

*Short Text Entry (Number of people)*

1. Please report how many total people you engaged with in the following activities over the *last month*:

4a. Around other people in the same physical space, but not interacting with

them: (e.g. in a coffeeshop, library, office building, bus, gym, waiting room, store,

etcetera)

4b. Interacting with others face-to-face

4c. Talking to one or more people on video calls such as Skype or FaceTime

**Results**

To examine the internal consistency of our mask exposure measure, the 11-items from the mask exposure section of the survey were z-scored, and the alpha was examined (α = 0.66). The contribution of individual items were examined, and the items with the lowest standardized correlation coefficient (std.r) were removed (4c, 4d, 5c, 5d). The alpha was recalculated with these items removed to create the final composite measure (α = 0.75).

To examine the internal consistency of our social interaction measure, the 8 items relating to in-person interaction from the social interaction section of the survey were z-scored, and the alpha was examined (α = 0.82).

To examine the internal consistency of our exploratory virtual social interaction measure, the 6 items relating to virtual interaction from the social interaction section of the survey were z-scored, and the alpha was examined (α = 0.63).
